# Acetyl-11-Keto-β-Boswellic Acid Accelerates the Repair of Spinal Cord Injury in Rats by Resisting Neuronal Pyroptosis with Nrf2

**DOI:** 10.3390/ijms25010358

**Published:** 2023-12-26

**Authors:** Yao Wang, Zongliang Xiong, Qiyuan Zhang, Mengmeng Liu, Jingjing Zhang, Xinyue Qi, Xiaowen Jiang, Wenhui Yu

**Affiliations:** 1 Department of Veterinary Medicine, Northeast Agricultural University, Harbin 150030, China; wangyao0116@hotmail.com (Y.W.); loveinchn@outlook.com (Z.X.); zhangqiyuan202210@163.com (Q.Z.); liumengmeng62022@163.com (M.L.); zjj816582@163.com (J.Z.); star99i@foxmail.com (X.Q.); 2 Key Laboratory of the Provincial Education Department of Heilongjiang for Common Animal Disease Prevention and Treatment, Northeast Agricultural University, Harbin 150030, China; 3 Institute of Chinese Veterinary Medicine, Northeast Agricultural University, Harbin 150030, China

**Keywords:** acetyl-11-keto-β-boswellic acid, spinal cord injury, Nrf2, NLRP3 inflammasome, neuronal pyroptosis

## Abstract

The primary aim of this study is to delve into the potential of Acetyl-11-keto-β-boswellic acid (AKBA) in ameliorating neuronal damage induced by acute spinal cord injury, as well as to unravel the intricate underlying mechanisms. A cohort of 40 Sprague-Dawley rats was meticulously categorized into four groups. Following a seven-day oral administration of AKBA, damaged spinal cord samples were meticulously procured for Nissl staining and electron microscopy to assess neuronal demise. Employing ELISA, immunofluorescence, Western blot (WB), and quantitative polymerase chain reaction (qPCR), the modulatory effects of AKBA within the context of spinal cord injury were comprehensively evaluated. Furthermore, employing an ex vivo extraction of spinal cord neurons, an ATP + LPS-induced pyroptotic injury model was established. The model was subsequently subjected to Nrf2 inhibition, followed by a battery of assessments involving ELISA, DCFH-DA staining, flow cytometry, immunofluorescence, and WB to decipher the effects of AKBA on the spinal cord neuron pyroptosis model. By engaging the Nrf2-ROS-NLRP3 pathway, AKBA exerted a repressive influence on the expression of the pyroptotic initiator protein Caspase-1, thereby mitigating the release of GSDMD and alleviating pyroptosis. Additionally, AKBA demonstrated the ability to attenuate the release of IL-18 and IL-1β, curbing neuronal loss and expediting the restorative processes within the context of spinal cord injury. Our study elucidates that AKBA can reduce spinal cord neuronal apoptosis, providing a basis for the development of AKBA as a clinical treatment for spinal cord injury.

## 1. Introduction

Spinal cord injury begets a cascade of perturbations in the local microenvironment, encompassing localized hemorrhage, stasis, edema, and thrombosis, leading to an intricate transformation [1]. These alterations in the microenvironment precipitate a rapid deterioration of microvasculature, thereby obstructing the spinal cord’s delicate ecosystem, impeding the essential supply of blood and oxygen, and exacerbating the tissue’s affliction [2]. Ischemic conditions trigger intracellular calcium overload, activating phospholipases, inducing peroxidation of membrane lipids, and degrading arachidonic acid, culminating in the synthesis of thromboxanes and leukotrienes. This sequence, in turn, fuels the escalation of oxygen free radicals and disrupts the electron transport chain within mitochondria, provoking an outpouring of free radicals. Such an upheaval catalyzes membrane lipid peroxidation, thereby engendering detriment to the structural integrity of both neurons and myelin sheaths, inevitably compromising their functionality [3].

Following spinal cord mechanical injury, a cascading neural inflammation ensues, setting the stage for a succession of secondary impairments that culminate in the demise of neurons. Furthermore, this cascade of secondary damage reactions incites an expansive inflammatory response within the injured region, thereby exacerbating the spinal cord injury (SCI). This inflammation extends its grip to adjacent tissues, significantly hampering axonal regeneration and functional recovery following SCI [4]. Present-day evidence underscores the pivotal role of the inflammasome signaling pathway in cellular pyroptosis, concurrently stimulating the secretion of an array of inflammatory factors [5,6]. Moreover, the process of neuroinflammation plays a critical role in the release and secretion of pro-inflammatory cytokines such as IL-1β and IL-18, thus initiating the mechanisms of cellular demise [7]. The activation of NLRP3 and the recruitment of apoptosis-associated speck-like protein containing a CARD (ASC) subsequently mediate Caspase-1 activation, orchestrating the maturation and secretion of pro-IL-1β and pro-IL-18 inflammatory cytokines [8]. Diverse stimuli, including reactive oxygen species (ROS), significantly trigger NLRP3 inflammasome and other inflammatory body constituents [9], and targeting NLRP3 and other inflammasome components notably enhances neuroprotective effects in murine models of SCI [10]. Caspase-1, in addition to its role in cytokine processing, cleaves the protein gasdermin D (GSDMD), thereby liberating its N-terminal domain (GSDMD-N). GSDMD-N, upon binding to the plasma membrane and oligomerization, forms pores that facilitate the release of mature IL-1β and IL-18 [11]. Throughout the course of cellular pyroptosis, the rupture of cell membranes, inclusive of the contents contained within inflammasomes, magnifies inflammatory reactions and exacerbates pathological responses. Thus, the cellular pyroptosis signaling pathway holds pivotal sway over central nervous system disorders [12]. NLRP3 is implicated in various neurological conditions, including instigating neuroinflammation following central nervous system injuries. The activation of NLRP3 inflammasomes triggers cellular pyroptosis and the subsequent release of inflammatory factors, thus amplifying neuroinflammation. Inflammatory bodies formed through the activation of classical Caspase-1 or non-classical Caspase-4/5/11-mediated pyroptotic pathways can, via intracellular and extracellular signaling pathways, incite inflammation, thereby further exacerbating the course of central nervous system disorders [13]. Existing research has demonstrated the critical role of NLRP3-mediated microglial cell pyroptosis in the inflammatory response during secondary spinal cord injury [14]. Hence, attenuating the exudation of inflammatory substances and cellular pyroptosis can shield spinal cord neurons from secondary damage, thus accelerating the process of spinal cord injury repair. Research suggests that betulinic acid, by quelling ROS and suppressing pyroptosis, facilitates the recuperation from spinal cord injury [15]. Furthermore, additional research underscores that CD73 exerts an expedited repair effect on spinal cord injury by inhibiting the activation of the NLRP3 inflammasome complex and consequently reducing the maturation of GSDMD, thereby suppressing microglial cell pyroptosis [16]. Oxidative stress refers to the elevation of intracellular ROS levels that lead to damage in lipids, proteins, and DNA. Studies indicate that mitochondrial ROS (mtROS) oxidizes four cysteine residues in GSDMD, promoting Caspase-1 cleavage of GSDMD, thereby augmenting the release of the GSDMD-N domain and cell death. Inhibiting the generation of ROS reduces the extent to which non-oxidized GSDMD is cleaved by Caspase-1, disrupting the enzymatic activity of Caspase-1 responsible for inflammasome activation consequently mitigating cytokine release and cellular demise [17]. Thus, inhibition of mtROS reduces GSDMD cleavage, thereby decreasing cellular pyroptosis. Furthermore, research indicates that upon activation of inflammasomes in Lrrk2G2019S macrophages, elevated mtROS guides the binding of the pore-forming protein GSDMD to the mitochondrial membrane, forming mitochondrial GSDMD pores. Subsequent release of mtROS accelerates the transition to RIPK1/RIPK3/MLKL-dependent necrosis, provoking heightened inflammation and severe immune pathology [18]. Therefore, ROS governs GSDMD to promote cellular pyroptosis. As ROS serves as an initiating factor for NLRP3, inhibiting ROS initiation exerts a restraining effect on NLRP3 activation, thereby diminishing cellular pyroptosis. Studies also propose that Prussian blue nanozyme (PBzyme) mitigates neurodegenerative lesions in Parkinson’s disease mouse models and cell models by clearing ROS and reducing the activation of microglial NLRP3 inflammasomes and Caspase-1. This consequently downregulates GSDMD cleavage, inhibits the production of inflammatory factors, and suppresses microglial cell pyroptosis [19].

Frankincense is the resin derived from the trees Boswellia carterii Birdw and Boswellia bhaurdajiana Birdw, belonging to the Oleaceae family. Acetyl-11-keto-β-boswellic acid (AKBA), present in the medicinal resin of frankincense, stands as a natural compound renowned for its pharmacological attributes, encompassing anti-inflammatory, antioxidant, and anti-tumor properties. Research studies have indicated that AKBA can diminish oxidative stress and alleviate neuroinflammation through the activation of Nrf2. Preliminary investigations by the project members have unveiled that AKBA can activate the ERK pathway, fostering Schwann cell proliferation to expedite the healing of sciatic nerve injury [20]. Moreover, it exerts its effects through phagocytosis and the secretion of neurotrophic factors [21], as well as by upregulating the expression of RhoA/Rictor to promote recovery from sciatic nerve injury [22]. However, the molecular mechanisms underlying the therapeutic action of AKBA in spinal cord injury remain elusive. Thus, this study has established both an in vivo model of AKBA treatment for spinal cord injury and an in vitro model of neuronal cell pyroptosis. Employing a spectrum of experimental techniques, including histopathology examination, immunofluorescence, Western blot, and real-time quantitative PCR, this research probes into the potential of AKBA in facilitating spinal cord injury repair and its underlying molecular mechanisms. This study aims to decipher whether AKBA can thwart neuronal pyroptosis, thus safeguarding the spinal cord against secondary damage, and to analyze the plausibility of AKBA as a therapeutic agent for spinal cord injury. Furthermore, it seeks to elucidate the intervention mechanism through which AKBA modulates spinal cord nerve injury, offering a foundation for the utilization of AKBA in treating spinal cord injury.

## 2. Results

### 2.1. AKBA Intervention Alleviates Neuronal Pyroptosis Caused by Spinal Cord Injury

**Figure 1:**
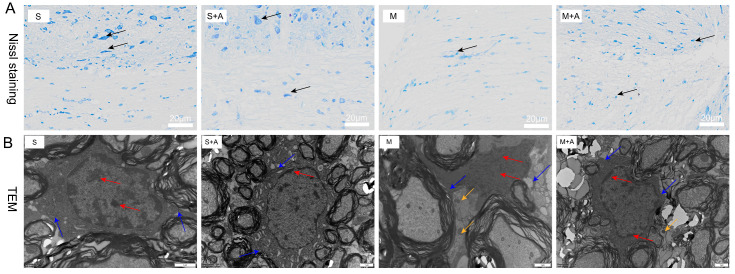
Effects of AKBA on Spinal Cord Neuronal Damage: Nissl Staining and Electron Microscopy Observations. (**A**) Nissl staining of spinal cord tissues. Dark blue staining in Nissl staining indicates neurons. Neurons are indicated by black arrows. The white scale bar represents 20 μm. (**B**) Electron microscopy observation of the impact of AKBA on rat spinal cord injury. Red arrows indicate chromatin and nucleoli, blue arrows point to cell membranes, and yellow arrows indicate inflammasomes. The black scale bar represents 1 μm. ‘S’ represents the sham-operated group, ‘S+A’ represents the sham-operated + AKBA group, ‘M’ represents the spinal cord injury model group, and ‘M+A’ represents the spinal cord injury model + AKBA group.

The Nissl bodies, vital sites of neuronal protein synthesis, exhibited significant reduction upon neuronal stimulation. As depicted in Figure 1A, neurons in the sham-operated group displayed large nuclei with convoluted nuclear membranes, sparse chromatin, and distinct nucleoli. Similarly, in the sham-operated + AKBA group, the neurons also exhibited substantial nuclei, sparse chromatin, and clear nucleoli. The neurons in the sham operation group and the sham operation + AKBA group were normal, and neurites were visible. In contrast, the model injury group showed neuronal cell atrophy and invisible neurites, indicating neuronal damage. Compared with the model group, although the same neuronal cell folds existed in the model group + AKBA group, neurites were visible, indicating that AKBA has a protective effect on spinal cord neuron damage. These findings collectively indicate that AKBA demonstrates neuroprotective effects by safeguarding the integrity of neurons.

To further elucidate the protective role of AKBA on neurons, this study employed electron microscopy to examine spinal cord tissues. During cellular pyroptosis, cells undergo swelling and develop protrusions on the cell surface prior to membrane rupture. Subsequently, pores appear on the cell membrane, causing cell disintegration, content release, inflammatory responses, nuclear condensation, and DNA fragmentation. As depicted in Figure 1B, neurons in the sham-operated group exhibited normal features with chromatin and nucleoli within the cell nucleus, intact cell membranes, and no release of inflammasomes. Similarly, the sham-operated + AKBA group showed no formation of inflammasomes compared with the sham-operated group. Conversely, the spinal cord injury model group displayed fragmented chromatin, lack of nucleoli, ruptured cell membranes, and release of inflammasomes. In contrast, the spinal cord injury model + AKBA group showed less pronounced chromatin fragmentation, ruptured cell membranes, and reduced release of inflammasomes. These results collectively indicate that AKBA can mitigate spinal cord cellular pyroptosis.

### 2.2. AKBA Alleviates Neuronal Pyroptosis-Related Pathways in Rat Spinal Cord Injury

**Figure 2:**
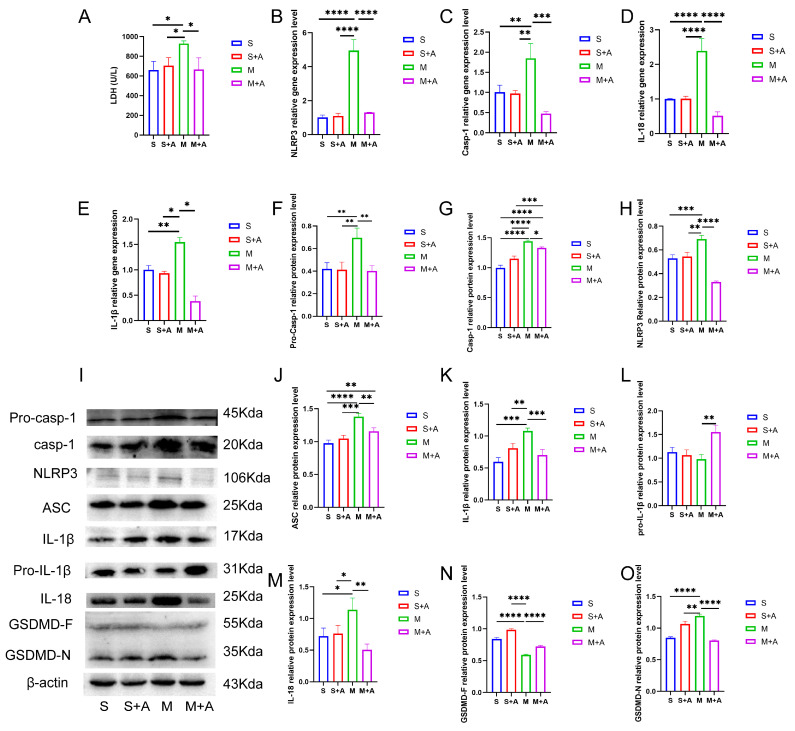
AKBA Attenuates Neuronal Pyroptosis-related Gene and Protein Expressions Post Spinal Cord Injury. (**A**) LDH release in spinal cord tissues. (**B**–**E**) Relative gene expression levels of NLRP3, Casp-1, IL-18, and IL-1β. (**F**–**H**) Relative protein expression levels of Pro-Casp-1, Casp-1, and NLRP3. (**I**) Protein expression overview of pyroptosis-related pathways. (**J**–**O**) Relative protein expression levels of ASC, IL-1β, Pro-IL-1β, IL-18, GSDMD-F, and GSDMD-N. * donated *p* < 0.05, ** donated *p* < 0.01, *** donated *p* < 0.001, and **** donated *p* < 0.0001.

Cellular pyroptosis leads to membrane rupture and the release of inflammatory substances. LDH is an important indicator of cell membrane integrity. As shown in Figure 2A, compared with the sham group, spinal cord injury (SCI) significantly increased LDH release (*p* < 0.05), while there was no significant difference in LDH release between the sham + AKBA group and the SCI model + AKBA group. Furthermore, the SCI + AKBA group exhibited significantly reduced LDH release compared with the SCI group (*p* < 0.05), suggesting that AKBA mitigates cell membrane rupture caused by spinal cord injury.

To further elucidate the protective effect of AKBA on neuronal pyroptosis, mRNA expression levels of pyroptosis-related genes (Casp-1, NLRP3, IL-18, IL-1β) were assessed. As depicted in Figure 2B,E, compared with the sham group, there was no significant difference in the mRNA expression of pyroptosis-related genes (Casp-1, NLRP3, IL-18, IL-1β) in the sham + AKBA group. However, the SCI group exhibited significantly elevated expression levels of these genes (*p* < 0.01 or *p* < 0.001). After AKBA intervention, the SCI + AKBA group demonstrated significantly decreased mRNA expression levels of pyroptosis-related genes compared with the SCI group. These results indicate that AKBA reduces the expression levels of pyroptosis-related genes.

To confirm the effect of AKBA on neuronal pyroptosis, protein expression levels of pyroptosis-related factors were examined. As shown in Figure 2F,O, NLRP3 expression significantly increased in the SCI group, whereas the SCI + AKBA group showed a marked reduction in NLRP3 expression compared with the SCI group. ASC expression significantly increased in the SCI group compared with the sham group, while AKBA intervention in the SCI + AKBA group resulted in a significant decrease in ASC expression compared with the SCI group. Casp-1 protein expression significantly increased in the SCI group compared with the sham group, but AKBA intervention attenuated this increase. Notably, AKBA effectively reduced the activation of Casp-1. GSDMD, a protein executing pore-forming cleavage activity at the N-terminus during cell pyroptosis, exhibited significantly higher expression in the SCI group compared with the sham group, while the SCI + AKBA group demonstrated a significant reduction in GSDMD-N expression compared with the elevated SCI group. IL-1β expression significantly increased in the SCI group compared with the sham group, but AKBA intervention resulted in a significant decrease in IL-1β expression compared with the SCI group. Similarly, IL-18 expression significantly increased in the SCI group compared with the sham group, yet the SCI + AKBA group exhibited a significant reduction in IL-18 expression compared with the SCI group. These findings collectively indicate that AKBA reduces NLRP3 expression, suppresses Casp-1 activation, decreases GSDMD-N expression, attenuates the release of IL-1β and IL-18, and alleviates cellular pyroptosis.

### 2.3. AKBA Mitigates Expression of Pyroptosis Initiation Protein

**Figure 3:**
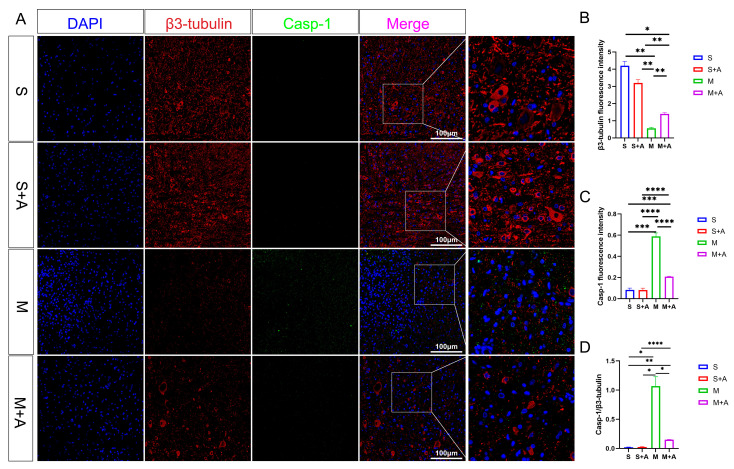
AKBA Attenuates Expression of Neuronal Pyroptosis Initiation Protein Casp-1. (**A**) β3-tubulin and Casp-1 immunofluorescence double staining images. (**B**) β3-tubulin relative fluorescence intensity. (**C**) Casp-1 relative fluorescence intensity. (**D**) Casp-1/β3-tubulin relative fluorescence intensity. * donated *p* < 0.05, ** donated *p* < 0.01, *** donated *p* < 0.001, and **** donated *p* < 0.0001.

To elucidate the state of neuronal pyroptosis, this experiment employed specific labeling of neurons using β3-tubulin and fluorescent labeling of the pyroptosis initiation protein, Casp-1. As depicted in Figure 3, both the sham surgery group and the sham surgery + AKBA group exhibited labeled neuronal proteins. In the spinal cord injury (SCI) model group, neuronal proteins were fragmented in the injured region, indicating neuronal damage. In the SCI + AKBA group, partial neuronal protein presence was observed in the injured region as well. Fluorescence intensity analysis of neuronal proteins revealed a significant reduction in β3-tubulin fluorescence intensity in the SCI model group compared with the sham surgery and sham surgery + AKBA groups. Conversely, compared with the SCI model group, the SCI + AKBA group showed a significant increase in β3-tubulin fluorescence intensity, although it remained significantly different from the sham surgery groups. Conversely, Casp-1 fluorescence intensity exhibited the opposite trend to that of β3-tubulin. Both compared with the sham surgery group and the sham surgery + AKBA group, the SCI model group displayed a significant upregulation of Casp-1 fluorescence intensity. In the SCI + AKBA group, Casp-1 fluorescence expression was significantly lower than that in the SCI model group. These results indicate that AKBA significantly reduces Casp-1 activation, thus safeguarding neurons. Additionally, the ratio of Casp-1 to β3-tubulin was evaluated. The results demonstrated a significant downregulation in the Casp-1/β3-tubulin ratio in the SCI + AKBA group compared with the SCI model group.

### 2.4. AKBA Attenuates Expression of Executory Pyroptotic Protein

**Figure 4:**
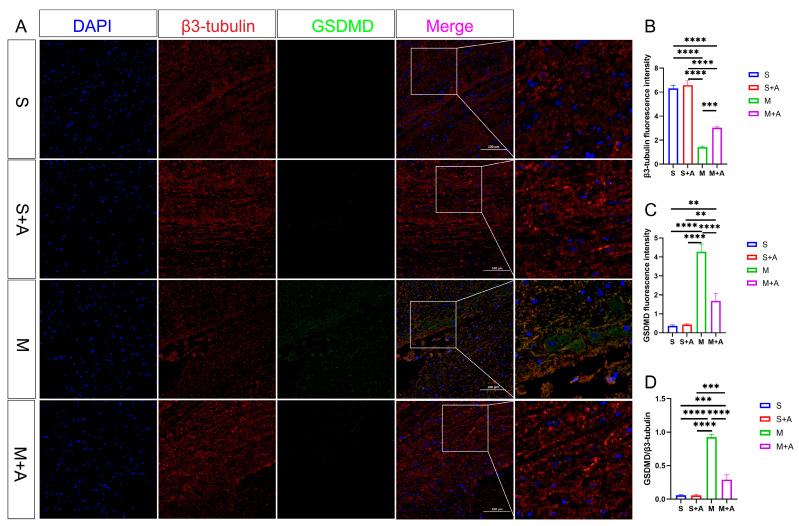
AKBA Attenuates Expression of Neural Pyroptotic Effector Proteins. (**A**) β3-tubulin and GSDMD immunofluorescence double staining images. (**B**) β3-tubulin relative fluorescence intensity. (**C**) GSDMD relative fluorescence intensity. (**D**) GSDMD/β3-tubulin relative fluorescence intensity. The ** donated *p* < 0.01, *** donated *p* < 0.001, and **** donated *p* < 0.0001.

In pursuit of a more nuanced understanding of neuronal pyroptosis, this investigation strategically harnessed β3-tubulin to delineate neuronal entities selectively. Concurrently, the execution protein GSDMD, instrumental in cellular pyroptosis, was judiciously fluorescently tagged. As eloquently depicted in Figure 4, both the sham surgery group and the sham surgery augmented with AKBA intervention unveiled distinct neuronal protein markers. However, within the realm of the spinal cord injury model, a conspicuous attenuation in neuronal protein materialization was noted at the lesion site. Notably, even though the spinal cord injury group followed by AKBA treatment exhibited a reduction in contrast to the injury group, a discernible elevation in protein expression was manifest vis-à-vis the spinal cord injury group. This discernible dichotomy reaffirms the protective mantle wielded by AKBA vis-à-vis spinal cord neurons. The GSDMD protein, a pivotal executor in the intricate narrative of cellular pyroptosis, exerts a potent impact on cellular membranes upon activation, consequentially releasing an arsenal of inflammatory mediators that further imperil neural integrity. Thus, the meticulous scrutiny of GSDMD assumes pivotal significance in dissecting the labyrinthine mechanisms at play. Fluorescence analysis, as meticulously expounded, revealed a substantial surge in GSDMD expression within the spinal cord injury group, diverging starkly from both the sham surgery group and the sham surgery augmented with AKBA. In intriguing contrast, the spinal cord injury group followed by AKBA treatment showcased a conspicuous dampening of fluorescence manifestation in comparison with its injury-affected counterpart. In addition, the calculated ratio between GSDMD and β3-tubulin underwent meticulous scrutiny. Impressively, the numerical formulation unveiled a marked decrement in the GSDMD/β3-tubulin ratio within the cohort representing spinal cord injury followed by AKBA intervention, in stark juxtaposition to the faction emblematic of solitary spinal cord injury. The amalgamation of these congruent findings unequivocally accentuates the efficacy of AKBA in orchestrating the abatement of GSDMD expression, thereby mitigating neural pyroptosis and conferring aegis upon the vulnerable neuronal ensemble.

### 2.5. AKBA Attenuates LDH Release and ROS Generation in a Spinal Neuronal Pyroptosis Model

**Figure 5:**
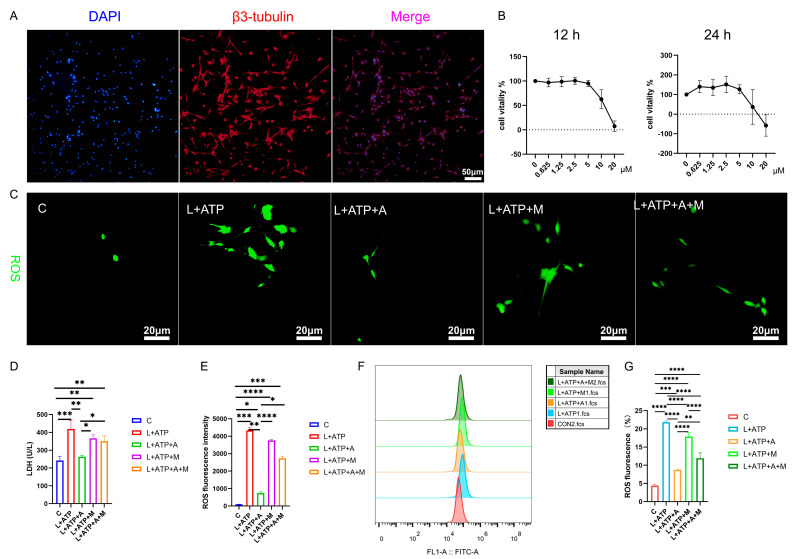
Illustrates the impact of AKBA on the expression levels of LDH and ROS in the spinal cord neuron apoptosis model. (**A**) β3-tubulin showcases the identification of neurons. (**B**) The determination of the optimal concentration of AKBA in the context of the spinal cord neuron apoptosis model. (**C**) The ROS release situation within the spinal cord neuron apoptosis model when subjected to AKBA intervention. (**D**) The expression pattern of LDH release under the influence of AKBA in the spinal cord neuron apoptosis model. (**E**) ROS fluorescence analysis chart. (**F**) ROS streaming results display chart. (**G**) ROS flow cytometry result analysis chart. “C” represents the blank control group, “L+ATP” represents the neuronal cell death model group, “L+ATP+A” represents the neuronal death model group with AKBA intervention group, “L+ATP+M” represents the neuronal death model group with Nrf2 inhibitor ML385 group, “L+ATP+A+M” represents the neuronal death model group with AKBA and Nrf2 inhibitor group. * donated *p* < 0.05, ** donated *p* < 0.01, *** donated *p* < 0.001, and **** donated *p* < 0.0001.

The intricate network of spinal neuronal integrity becomes exquisitely discernible through the lens of this scientific exploration. Transcending mere observation, this study navigates the realm of neuronal vitality, guided by the beacon of β3-tubulin, a poignant marker of neuronal identity. Embodied within Figure 5A, the resplendent overlap of β3-tubulin within primary neuronal extracts attains a striking congruence, surpassing the threshold of 90%. This harmonious convergence not only signifies neuronal authenticity but also lays the foundation for subsequent investigative strides.

Further transcending the boundaries of observation, the researchers’ discerning gaze turns towards the labyrinth of cellular viability, as unveiled in Figure 5B. Through a symphony of LPS stimulation and AKBA intervention, the optimal dosage and duration of administration are meticulously delineated. The orchestrated interplay of factors reveals the resonance of neuronal vigor at the 2.5 μM threshold over 12 h, a testament to the exquisite choreography of cellular responsiveness under the guardianship of AKBA.

The intricate web of cellular balance is poignantly unveiled as this study delves into the nexus of pyroptotic mechanics. The elusive dance of cell membrane integrity finds expression through the release of lactate dehydrogenase (LDH), a sentinel of cellular wholeness. Figure 5D resonates with the echoes of this cellular narrative: the ascent of LDH release in the L+ATP ensemble is met with the tempering grace of the presence of AKBA. Yet, the saga takes an intriguing twist as the intervention of ML385 emerges, dampening the cascade of LDH suppression orchestrated by AKBA. This intricate ballet of influences culminates in a revelation—the ebb and flow of LDH liberation are underpinned by the profound regulatory sway of AKBA.

In the realm of cellular dynamics, the omnipresent specter of reactive oxygen species (ROS) heralds both vitality and vulnerability. Figure 5C,E unravels the intricate dialogue between AKBA and ROS within the spinal neurons. As L+ATP orchestrates the surge of ROS expression, the intervention of AKBA emerges as a sentinel of equilibrium, orchestrating a profound reduction in ROS orchestration catalyzed by LPS and ATP. Yet, the crescendo of control attains its zenith as ML385 takes the stage, further modulating the trajectory of ROS expression under the tutelage of AKBA. Thus, the narrative unfurls—AKBA, guided by the conduit of Nrf2, emerges as a beacon of restraint, harmonizing the symphony of ROS release in the intricate tapestry of neuronal resilience. We also tested the proportion of ROS through flow cytometry and obtained the same results as the staining (Figure 5F,G). Compared with the blank group, the proportion of ROS in the L+ATP group was significantly increased, which further demonstrated the success of the established model and adding AKBA can significantly reduce the proportion of ROS, but after adding the Nrf2 inhibitor ML385, the proportion of AKBA reducing ROS was inhibited. Therefore, it is shown that AKBA can significantly reduce the activity of ROS.

In this symposium of scientific exploration, each figure weaves a narrative of cellular nuances, unraveling the mysteries of the guardianship of AKBA over neuronal vitality. As we traverse the corridors of inquiry, we glimpse the promise of the intervention of AKBA, orchestrating a dance of equilibrium within the intricate ensemble of spinal neurons.

### 2.6. Impact of AKBA on Mitochondrial Membrane Potential in Rat Spinal Neurons

**Figure 6:**
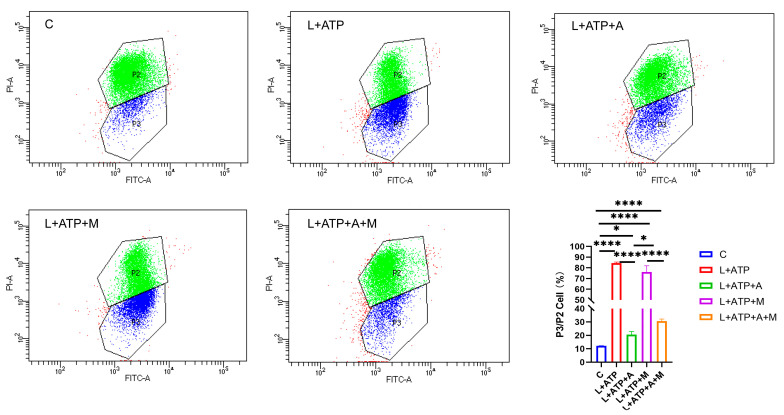
Influence of AKBA on Mitochondrial Impairment in Spinal Neurons. P2 represents the number of cells with red fluorescence (PI-A), and composed of green dots in the figure. P3 represents the number of cells with green fluorescence (FITC-A), and composed of blue dots in the figure. * donated *p* < 0.05, and **** donated *p* < 0.0001.

In order to elucidate the effect of AKBA on neuronal pyroptosis, meticulous exploration was undertaken to discern its influence on mitochondrial health within the spinal neuronal milieu. In this intricate narrative, the crescendo of LPS and ATP assumes the role of instigators, leading to the orchestration of spinal neuronal injury. The narrative unfolds through the lens of JC-1, casting light upon the subtle variations in mitochondrial membrane potential across different treatment paradigms, as eloquently depicted in Figure 6. JC-1, the messenger of this saga, manifests its presence through two distinct ensembles, each bearing testament to the ebb and flow of mitochondrial vitality. Within the ethereal realm of higher mitochondrial membrane potential, JC-1 converges, yielding an ensemble that orchestrates a symphony of red fluorescence (PI-A), a visual ballet of cellular health. However, the wane of mitochondrial vitality, portrayed by lower membrane potential, begets a transformation wherein the presence of JC-1 shifts to a monomeric form, radiating an enchanting green fluorescence (FITC-A). Herein, the essence lies—a reduced membrane potential foretells the chalice of cellular distress. Through the prism of this spectral interplay, the quest for a balanced equilibrium is woven. The analysis of the ratio between green and red fluorescence heralds the degree of cellular injury. As the tableau unfolds, it becomes evident that within the realm of L+ATP, a crescendo of mitochondrial harm emerges, juxtaposed against the contrasting backdrop of the intervention of AKBA. The narrative deepens as ML385, the harbinger of inhibition, strides onto the scene, shaping the trajectory of the mitigatory influence of AKBA on mitochondrial integrity. Relative to the hallowed sanctuary of the control group (C), the orchestration of mitochondrial damage becomes amplified across experimental realms. While AKBA emerges as a virtuoso, skillfully attenuating the swathes of damage inflicted by L+ATP, it is not without its own thresholds. The introduction of ML385 engenders a medley of inhibition, tempering the power of AKBA to assuage the choir of mitochondrial harm. As the grand tapestry unfurls, AKBA emerges as a beacon of promise, diminishing the echoes of mitochondrial distress. Yet, the complexities remain, as the boundary of complete inhibition eludes even the deft touch of AKBA. This symphony, depicted in Figure 6, narrates the odyssey of the mastery of AKBA over mitochondrial vitality, a journey of modulation and inhibition guided by the hand of science and conducted by the tendrils of nature’s orchestration.

### 2.7. The Impact of AKBA on the Expression of Crucial Proteins Associated with Spinal Neuronal Pyroptosis

**Figure 7:**
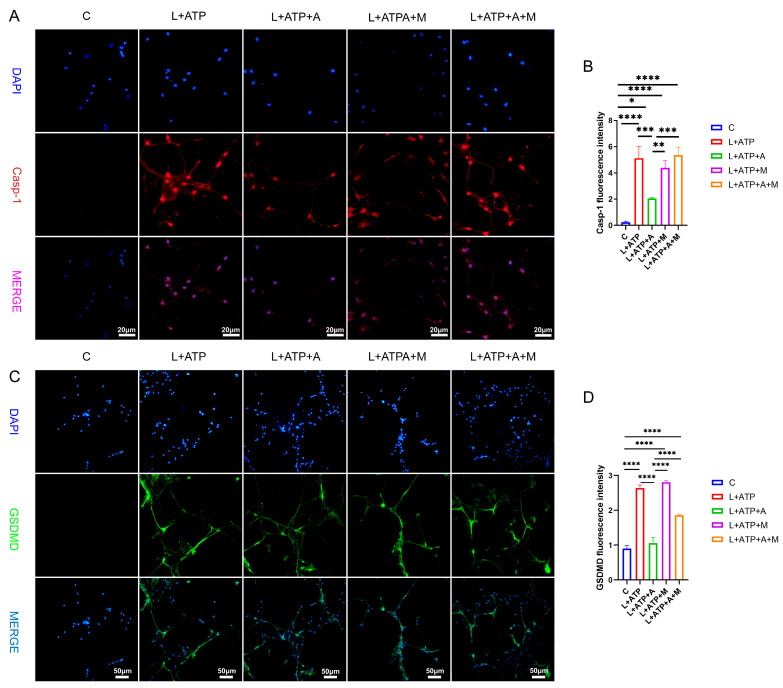
The Impact of AKBA on Crucial Proteins Associated with Spinal Neuronal Pyroptosis. (**A**) Fluorescent Expression Mapping of Casp-1 in Spinal Neurons Undergoing Pyroptotic Model in Response to AKBA. (**B**) Relative Fluorescent Expression Levels of Casp-1. (**C**) Fluorescent Expression Mapping of GSDMD in Spinal Neurons Undergoing Pyroptotic Model in Response to AKBA. (**D**) Relative Fluorescent Expression Levels of GSDMD. * donated *p* < 0.05, ** donated *p* < 0.01, *** donated *p* < 0.001, and **** donated *p* < 0.0001.

The expression of key regulatory protein Casp-1 within the canonical pathway of cellular pyroptosis plays a pivotal role in the initiation of cellular pyroptosis. Hence, this experimentation entailed the assessment of Casp-1 expression levels within spinal neurons. As illustrated in Figure 7A,B, comparative analysis with the Control (C) group revealed a significant upregulation of Casp-1 expression levels in the L+ATP, L+ATP+A, L+ATP+M, and L+ATP+A+M groups. Notably, a marked downregulation of Casp-1 expression was observed in the L+ATP+A group compared with the L+ATP group, whereas no significant variance was evident between the L+ATP+M and L+A+ATP+M groups. These findings signify the substantial potential of AKBA to attenuate the expression of Casp-1 elicited by L+ATP, thereby mitigating pyroptotic events. It is noteworthy, however, that the introduction of the Nrf2 inhibitor curbed the diminishing effect of AKBA on Casp-1 expression levels. The results collectively illuminate that while AKBA possesses the capacity to mitigate the expression of Casp-1 appreciably, this capacity is counteracted by the presence of the Nrf2 inhibitor ML385.

To elucidate the role of AKBA in spinal neuronal pyroptosis, a fluorescent assessment of gasdermin D (GSDMD), an executioner protein of pyroptosis, was conducted. As depicted in Figure 7C,D, discernible upregulation of GSDMD fluorescence was manifest in the L+ATP, L+ATP+A, L+ATP+M, and L+ATP+A+M groups in comparison with the Control (C) group. Conversely, the L+ATP+A group exhibited a significant downregulation in GSDMD expression levels as contrasted with the L+ATP group. Yet, the introduction of the Nrf2 inhibitor significantly mitigated the trend of AKBA-induced downregulation. Collectively, these observations suggest that AKBA orchestrates modulation of GSDMD expression through Nrf2 regulation.

### 2.8. The Influence of AKBA on Spinal Neuronal Pyroptosis-Associated Proteins

**Figure 8:**
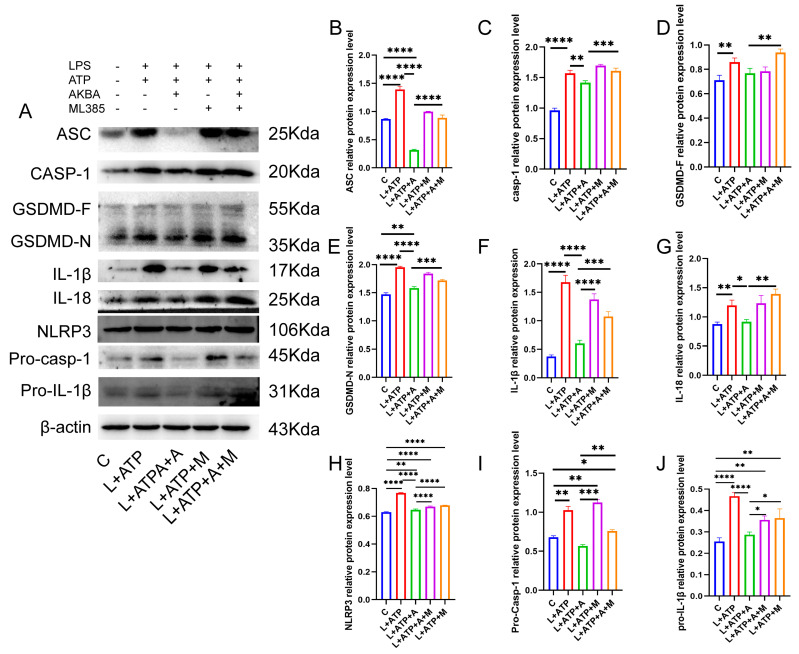
AKBA Mitigates Protein Expression Levels Associated with Spinal Neuronal Pyroptosis. (**A**) Protein Expression Mapping of Pyroptosis-Associated Pathway Proteins. (**B**) Quantitative Analysis of ASC Protein. (**C**) Quantitative Analysis of Casp-1 Protein. (**D**) Quantitative Analysis of GSDMD-F Protein. (**E**) Quantitative Analysis of GSDMD-N Protein. (**F**) Quantitative Analysis of IL-1β Protein. (**G**) Quantitative Analysis of IL-18 Protein. (**H**) Quantitative Analysis of NLRP3 Protein. (**I**) Quantitative Analysis of Pro-Casp-1 Protein. (**J**) Quantitative Analysis of Pro-IL-1β Protein. * donated *p* < 0.05, ** donated *p* < 0.01, *** donated *p* < 0.001, and **** donated *p* < 0.0001.

The NLRP3 inflammasome pathway, a canonical driver of cellular pyroptosis, was interrogated in this experiment through the assessment of pertinent proteins. As illustrated in Figure 8, comparative analysis with the Control (C) group revealed a pronounced upregulation in the expression levels of the adaptor protein ASC within the NLRP3 inflammasome in the L+ATP group. The subsequent introduction of AKBA elicited a discernible restraint on the escalating trend of expression. This restraint signifies a reduction in the synthesis of the NLRP3 inflammasome. Furthermore, in comparison with the Control (C) group, the expression levels of Pro-Casp-1 and Casp-1 exhibited significant elevation in the L+ATP group. However, juxtaposition with the L+ATP+A group demonstrated that the introduction of AKBA curbed the upregulation of Casp-1. Consequently, the activation of Casp-1 resulted in the inhibition of the activity of the GSDMD-N terminus. Subsequent to the curbing of GSDMD-N terminus activity, downstream inflammatory factors IL-18 and IL-1β were notably suppressed.

Nevertheless, the inclusion of the Nrf2 inhibitor relieved the suppression of the NLRP3 inflammasome, Casp-1, GSDMD-N, as well as IL-18 and IL-1β. Collectively, these observations underscore the potential of AKBA to mitigate cellular pyroptosis by curtailing the expression of GSDMD-N through the inhibition of the NLRP3/Casp-1 axis, thereby fostering neuroprotection within neuronal cell populations.

## 3. Discussion

This study’s results highlight the impact of AKBA on mitigating spinal neuronal pyroptosis-associated phenomena. Neuronal Nissl bodies, present in triangular or elliptical formations within the cytoplasm, exhibit distinct purple-blue staining with basic dyes such as toluidine blue or Nissl stain [23]. These structures are indicative of neuronal health, and their presence or absence serves as a crucial indicator of cellular damage [24]. The current experiment demonstrates that AKBA intervention offers protection to spinal neurons when compared with the spinal injury model group. To further elucidate the neuroprotective mechanism of AKBA, electron microscopy observations of spinal tissue revealed its ability to attenuate neuronal pyroptosis and thereby safeguard neurons effectively. Cellular pyroptosis often involves cell membrane rupture, leading to the release of lactate dehydrogenase (LDH) [25,26]. The observed reduction in LDH expression upon AKBA intervention compared with the spinal injury group underscores the ability of AKBA to mitigate cellular pyroptosis. To delve deeper into the role of AKBA in spinal neuronal pyroptosis, this study dual-stained classic pyroptosis initiator Casp-1 along with the neuronal marker β3-tubulin. In the spinal injury model group, Casp-1 expression was markedly upregulated, while β3-tubulin expression and distribution were significantly reduced, indicating neuronal loss due to pyroptosis. However, in the spinal injury model + AKBA group, Casp-1 expression decreased, and β3-tubulin expression increased, suggesting less severe neuronal loss. This reaffirms that pyroptosis-induced neuronal loss contributes to secondary spinal injuries. This study also investigated whether Casp-1 activation correlates with the executioner protein GSDMD. Immunofluorescence revealed significant upregulation of GSDMD in the spinal cord injury model group, which was attenuated by AKBA intervention. This further corroborates the potential of AKBA to diminish cellular pyroptosis and protect spinal tissue. Subsequently, Western blot analyses were conducted on various pyroptosis-related proteins, including NLRP3, ASC, Pro-IL-β, Pro-Casp-1, IL-1β, IL-18, GSDMD, and Casp-1. AKBA demonstrated the capacity to attenuate protein expressions linked to NLRP3, ASC, Casp-1, and GSDMD-N elevation caused by spinal injury. Consequently, this reduction contributed to diminished expression levels of the inflammatory substances IL-1β and IL-18, effectively curbing cellular pyroptosis and safeguarding neurons from injury induced by spinal trauma. In conclusion, this study elucidates the potential of AKBA in attenuating spinal neuronal pyroptosis-associated processes. The intervention of AKBA demonstrated the ability to mitigate neuronal loss, suppress inflammatory factors, and reduce cellular pyroptosis, thus offering neuroprotection against secondary damage resulting from spinal injuries. These findings could have significant implications for the development of therapeutic strategies targeting pyroptosis-mediated neurodegenerative disorders.

Post-spinal cord injury, an increase in reactive oxygen species (ROS) production occurs [27], followed by oxidative stress, inflammasome formation, pyroptosis, and inflammatory responses [10,28]. Cellular pyroptosis leads to the release of inflammatory cytokines such as IL-18 and IL-1β [29], exacerbating neural inflammation and hindering spinal cord recovery [7]. Prior research studies indicate that pre-treatment with baicalein reduces secondary spinal cord injury-induced pyroptosis by inhibiting the MAPKs-NF-κB signaling pathway, thereby protecting spinal tissue [30]. Another study demonstrates that advanced oxidized protein products (AOPPs), a marker of oxidative stress, increase in SCI models and contribute to inflammation via ROS-dependent MAPK-NF-κB and NLRP3-GSDMD signaling pathways [31]. Inhibiting NADPH oxidase with apocynin suppresses AOPPs-induced oxidative stress, subsequently decreasing ROS production, inflammation, and pyroptosis [31]. Inhibition of SARM1 has been shown to suppress NF-kB, thereby reducing neural inflammation and promoting spinal neuron regeneration [32]. Additionally, ROS positively regulates GSDMD pore formation [33]. AKBA, the intervention used in this study, has been shown to decrease ROS production through Nrf2 activation [34,35]. Based on this, this study hypothesized that AKBA could reduce ROS expression via Nrf2, thereby decreasing NLRP3-GSDMD expression levels and subsequently inhibiting pyroptosis, ultimately protecting the spinal cord from injury. To validate this hypothesis, primary spinal neurons were extracted and exposed to LPS and ATP to induce a pyroptosis model [30,36]. The addition of Nrf2 inhibitor ML385 was employed to explore the impact of AKBA on spinal neuronal pyroptosis. LDH release, a marker of cell membrane rupture, was measured [37]. Results indicated that AKBA reduced LDH release compared with the LPS + ATP group, while the addition of ML385 suppressed this reduction. Since ROS can positively regulate NLRP3 and GSDMD expressions, ROS levels were assessed [38,39]. ROS expression was significantly upregulated in the LPS + ATP pyroptosis model group, while AKBA reduced ROS expression. ML385 mitigated the suppressive effect of AKBA. This suggests that AKBA reduces ROS expression through Nrf2. JC-1 staining revealed that AKBA reduced mitochondrial membrane potential damage in the LPS + ATP pyroptosis model group, with ML385 attenuating this effect. The experiment inferred that AKBA regulates mitochondrial damage through the Nrf2/ROS pathway. Mitochondrial membrane potential damage induces ROS release, further activating downstream NLRP3 inflammasome, which ultimately leads to Casp-1 activation and pyroptosis initiation [40]. Casp-1 fluorescence staining indicated that AKBA reduced Casp-1 expression, subsequently dampening the activation of the pyroptosis pathway. This study then observed GSDMD, the effector protein of pyroptosis [41], and found that AKBA decreased GSDMD expression levels. The protein expression levels of ASC, NLRP3, Casp-1, Pro-Casp-1, Pro-IL-1β, GSDMD, IL-1β, and IL-18 were tested. AKBA significantly reduced the expressions of ASC, NLRP3, Casp-1, and GSDMD-N, leading to decreased IL-1β and IL-18 activation. This curtailed pyroptosis, reduced inflammatory substance leakage, and safeguarded spinal neurons from pyroptosis-induced damage. Existing research on dihydromyricetin (DHM) aligns with the results of this experiment. DHM eliminates ROS and cell pyroptosis induced by palmitic acid through Nrf2 activation. Lowering Nrf2 with siRNA eliminates the inhibitory effects of DHM, indicating the ability of DHM to eliminate ROS and reduce cell pyroptosis [42]. This is consistent with the ability of AKBA, demonstrated in this study, to reduce ROS and cell pyroptosis through Nrf2 activation.

**Figure 9:**
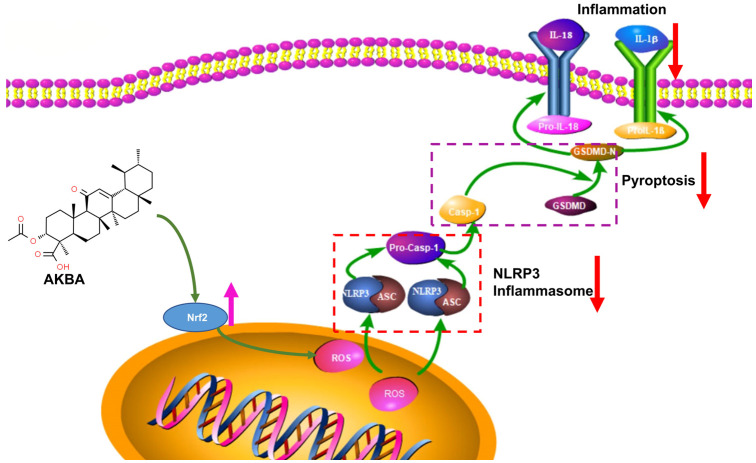
Mechanism of Action of AKBA.

In conclusion, the findings of this study underscore the ability of AKBA to upregulate Nrf2 expression, leading to reduced ROS production. This subsequently diminishes NLRP3 activation, inhibits Casp-1 activation, and reduces GSDMD expression. Collectively, these effects culminate in the mitigation of cellular pyroptosis, leading to a reduction in the release of inflammatory substances and ultimately providing neuroprotection to neurons (refer to Figure 9).

## 4. Materials and Methods

### 4.1. Construction and Administration of Spinal Cord Injury Model

Forty female Sprague-Dawley rats, aged 8 weeks and weighing 180–200 g, were procured from Liaoning Changsheng Biotechnology Company for experimentation. The spinal cord injury model was established using the technique of arterial clamp compression [43] to ensure consistency in the injury induction. The experimental protocol received approval from the Animal Ethics Committee of Northeast Agricultural University, with approval number NEAUEC20220508. Rats were provided with ad libitum access to food and tap water and were maintained under controlled conditions of 12 h light/dark cycles at a temperature of 21 ± 1 °C.

The meticulous procedures, including ethical approval, animal welfare, and standardized model construction, collectively contribute to the ethical integrity and scientific rigor of animal experimentation. These measures ensure that the research is carried out responsibly and in adherence to ethical guidelines. Once the rats were adequately anesthetized by Zoletil-50, standard surgical preparation was carried out. The skin was transversely incised at the level of the thoracic vertebrae (T6-T7) at the surgical site, and the muscle tissue was dissected. Utilizing fine scissors and delicate forceps, a laminectomy was performed at the T6-T7 vertebrae. During this step, care was taken to ensure the integrity of the dura mater. A section of the facet joint was carefully removed to create sufficient space for the insertion of an aneurysm clip, which was utilized for compressing the spinal cord. The spinal cord was compressed once using the aneurysm clip for a duration of 1 min, adhering to the procedure described [43]. Upon the completion of spinal cord compression modeling, the muscle and skin layers were meticulously sutured separately. Following the surgery, all rats were placed in isolation cages under controlled temperature conditions for a period of 2–3 h. In cases of bladder dysfunction, even though symptoms may be atypical after a compression injury, bladder massage was performed twice daily until normal bladder function was restored. It is important to note that the sham operation group rats underwent the same surgical steps as the model group rats, including anesthesia, skin and muscle incision, and laminectomy. However, the actual spinal cord compression step was omitted for the sham operation rats. This careful procedure design ensures uniformity and comparability across the experimental groups while replicating surgical conditions.

After the completion of the surgery, the experimental groups were as follows: Sham operation group (S), Sham operation with AKBA group (S+A), Spinal cord injury model group (M), and Spinal cord injury model with AKBA group (M+A). Each group consisted of 10 rats. The treatment groups received daily oral administration of AKBA at a dosage of 20 mg/kg [44]. The control groups were administered an equivalent volume of solvent via oral gavage.

The administration duration was 7 days. AKBA weighing 80 mg was dissolved in 1 mL of DMSO (80 mg/mL) to create a stock solution. This stock solution was further diluted to create an oral gavage solution with a proportion of 4 mg/mL using the solvent mixture (5% DMSO + 30% polyethylene glycol 300 + 65% distilled water). The rats were orally administered the drug at a dose of 0.5 mL/100 g body weight. For the solvent control group, the oral gavage solution consisted of the solvent mixture without AKBA (5% DMSO + 30% polyethylene glycol 300 + 65% distilled water). The precise administration protocol and the formulation of the drug and control solutions are pivotal to ensuring accurate dosing and maintaining consistency across experimental groups. This meticulous approach ensures that the effects observed can be attributed to the specific intervention and dosage.

### 4.2. Nissl Staining for Neuronal Loss Observation

Rats were euthanized through cervical dislocation following anesthesia with Zoletil-50. The spinal cord at the injury site was fixed using 4% paraformaldehyde to create paraffin-embedded sections. These paraffin sections underwent dewaxing and water washing using the following steps: sections were immersed in xylene I for 20 min, followed by xylene II for 20 min, absolute ethanol I for 5 min, absolute ethanol II for 5 min, and finally, 75% alcohol for 5 min. The sections were then rinsed with tap water. Subsequently, a toluidine blue staining solution was applied dropwise. After allowing the staining process to occur at room temperature for 45 min, the sections were rinsed with water. Aniline blue staining was performed by immersing the sections in the aniline blue staining solution for 5–10 min, followed by rinsing with tap water. The sections were then placed in an oven set at 60 °C for drying. Dehydration and sealing steps followed: the sections were placed in xylene for 5 min to achieve transparency, and then they were sealed with neutral gum. Following these procedures, the sections were subjected to microscopic examination, with images acquired for subsequent analysis. This comprehensive procedure, from tissue processing to staining and microscopic analysis, provides a detailed insight into the morphological changes occurring within the spinal cord tissue, enabling a thorough assessment of the impact of the experimental interventions.

### 4.3. Ultrastructural Observation of Cellular Apoptosis

Rats were sacrificed by cervical dislocation after anesthesia with Zoletil-50, and the spinal cord tissue at the injured site was fixed with 2.5% glutaraldehyde for 10 min. Tissues were removed from each group of fixed tissues, cut into cubes with a side no larger than 1 mm, and then placed in fixative for 7 days. After being fixed, it was washed and dehydrated with ethanol and then resin-embedded within resin, then ultra-thin sectioned, stained, photographed using a transmission electron microscope, and analyzed for image data.

### 4.4. Immunofluorescence Analysis of Casp-1 and GSDMD Protein Expression

The following steps were undertaken for the immunofluorescence staining of paraffin sections: Paraffin-embedded sections were deparaffinized by sequential immersion in xylene and decreasing concentrations of ethanol. Sections were subjected to antigen retrieval by placing them in a 0.01 M citrate buffer (pH 6.0) in an antigen retrieval box and heating them in a microwave for 10 min. This step aids in exposing antigens for antibody binding. Sections were dried slightly and then circled using a hydrophobic pen. A blocking solution (such as BSA) was added to the sections to minimize non-specific binding. The sections were incubated with primary antibodies specific to β3-tubulin (1:200, ABclonal, Wuhan, China), Casp-1 (1:200, Wanleibio, Shenyang, China), or GSDMD (1:200, Affinity, Shanghai, China) overnight at 4 °C. After washing off unbound primary antibodies, appropriate secondary antibodies labeled with fluorescent dyes (such as CY-3 for β3-tubulin and FITC for Casp-1/GSDMD) were added to the sections. The sections were then subjected to microwave treatment to enhance antibody binding. DAPI staining was performed to label cell nuclei. A fluorescence quenching reagent was added to minimize background fluorescence. Finally, the sections were mounted and coverslipped. The immunofluorescently stained sections were examined under a fluorescence microscope. The specific fluorescence emitted by labeled proteins and nuclei was visualized and captured. This methodology allows for the visualization of β3-tubulin, Casp-1, and GSDMD protein expression within the spinal cord tissue. The distinct fluorescence of the labeled antibodies highlights the presence and distribution of these proteins, contributing to the understanding of their roles in spinal cord injury and the potential impact of AKBA treatment. For immunofluorescence pictures, we randomly selected five 200× fields of view and then analyzed the fluorescence intensity through Image-J. The relative fluorescence intensity of the target protein is determined by the fluorescence intensity of the target protein/the fluorescence intensity of DAPI.

### 4.5. Lactate Dehydrogenase Release Assay

The determination of LDH release was carried out in strict accordance with the instructions of the LDH kit.

### 4.6. Western Blot of NLRP3 Pyroptotic Pathway-Associated Proteins

Spinal cord tissue (*n* = 3) was extracted following the protocol outlined by Xiong et al. [45]. Afterward, a BCA assay was employed to determine protein concentrations, which were then standardized. Utilizing the standardized protein, the 5× protein loading buffer was diluted to 1×. Subsequently, protein gels were prepared, with each well loaded with 30 μg of sample. Electrophoresis was initiated at 80 V for the upper gel, maintaining this condition for 30 min, followed by a transition to 120 V until electrophoresis completion. Following gel excision and transfer onto PVDF membranes, a rapid sealing solution was applied. An overnight incubation with the diluted primary antibody (antibody dilution as per Table 1) was conducted after blocking. On a subsequent day, after washing the PVDF membranes, they were subjected to a 2 h incubation with goat anti-rabbit horseradish peroxidase (HRP)-conjugated secondary antibody (1:10,000, Bioss, Beijing, China). Following further washing, a highly sensitive ECL chemiluminescence reagent was applied. Subsequently, the membranes were exposed using the Tanon 5200 automated chemiluminescence imaging system. Image data were subjected to analysis using ImageJ software (fiji 2.15.0), and the results were presented as the ratio of target protein to β-actin.

### 4.7. Quantitative Real-Time PCR for Analysis of NLRP3 Pyroptotic Pathway-Related Genes

Spinal cord tissue(*n* = 3) was subjected to total RNA extraction according to the protocol outlined by Chi et al. [46]. Subsequently, the RNA concentration was determined, with a 260/280 ratio within the range of 1.8–2.0 considered acceptable. The extracted RNA was then subjected to reverse transcription using a reverse transcription reagent kit, yielding complementary DNA (cDNA). Primers for the target genes were designed using Primer Blast and synthesized by a specialized biotechnology company. The qPCR primers were designed using NCBI, and their specificity was verified in BLAST. The primer sequences are detailed in Table 2. The PCR system was prepared according to the manufacturer’s instructions, and the prepared mixture was added to a 96-well Roche PCR plate. After thorough mixing, the plate was placed into a Roche 480 real-time quantitative fluorescence PCR instrument. The program settings were in accordance with the dye’s specifications. Data analysis employed the ΔΔCT method, with β-actin serving as the internal reference. The sham operation group was designated as the control. The relative expression level was calculated using the formula 2^−∆∆CT^, where ∆CT represents the difference between the target gene’s cycle threshold (CT) and the reference gene’s CT for each sample, and ∆∆CT represents the difference in ∆CT values between the treated and control groups.

### 4.8. Isolation of Spinal Cord Neurons from Rats

Extraction of spinal cord neurons from rats was conducted following the methodology outlined by Huang et al. [47]. Briefly, rats were immersed in 75% ethanol for a brief moment, and after decapitation using scissors, the rodents were bled to remove residual blood. The thoracic vertebra T10 was utilized to remove spinous processes and laminae carefully using micro bone forceps, followed by the opening of the dura mater. A thorough washing of the spinal cord tissue to eliminate any remaining blood was then performed. Subsequently, the spinal cord tissue was sectioned into small fragments and transferred into sterile centrifuge tubes containing 0.25% EDTA–trypsin, where gentle aspiration was carried out 15–20 times. The digestion was halted within a laminar flow hood using 2 mL of neuron culture medium containing 10% FBS. Tissue homogenate was then filtered through a 70 μm cell strainer, and the filtrate was collected into sterile centrifuge tubes. Centrifugation at 1000 rpm for 5 min followed, with the supernatant discarded and replaced by neuron culture medium supplemented with 10% fetal bovine serum (FBS) to create a single-cell suspension. The cell concentration was adjusted to 1 × 10^6^ cells/mL. Subsequently, the cell suspension was seeded into cell culture dishes, with 500 μL per well in a 24-well culture plate. Cultivation was carried out at 37 °C under 5% CO_2_ for 24 h, with media changes every day. After three days, arabinofuranosyl cytidine (1 μM) was introduced to inhibit the proliferation of non-neuronal cells. Neuronal growth was observed daily over the course of cultivation.

### 4.9. Establishment of Spinal Cord Neuronal Pyroptosis Model and Screening for Optimal AKBA Dosage

The composition of the neuron culture medium consisted of a Neurobasal medium supplemented with 2% B27 and 1% L-glutamine. Utilizing the aforementioned methodology, primary spinal cord neurons were extracted. Subsequently, the cells were categorized into five groups: Control (C) group, LPS + ATP (L+ATP) group, LPS + ATP + AKBA (L+ATP+A) group, LPS + ATP + ML385 (L+ATP+M) group, and LPS + ATP + AKBA + ML385 (L+ATP+A+M) group. The establishment of the pyroptosis model followed the protocol outlined by Han et al., involving an initiation with 100 ng/mL of LPS for 24 h, succeeded by a 30 min stimulation with 5 mM ATP [48]. Additionally, the determination of the optimal AKBA dosage was achieved through the employment of the CCK-8 assay. During the administration of AKBA, varying concentrations were concurrently introduced to the model. The viability of the cells was assessed to ascertain the most suitable drug concentration. In the experimental setup, the C group served as the blank control group. For the remaining groups, the addition of the drug involved exchanging the medium without adding the drug. The dosage of ML385 administration was set at 5 μM.

### 4.10. Immunofluorescence Detection of Caspase-1 and GSDMD Expression in Cells

Following cell cultivation, fixation was accomplished using 4% paraformaldehyde for 15 min, followed by a triple wash with PBS. Subsequently, permeabilization was achieved by adding 0.25% Triton X-100 for 20 min, followed by another triple PBS wash, each lasting 5 min. A serum-blocking step of 30 min ensued, after which the blocking solution was aspirated, and the cells were washed gently and dried. Then, after carefully applying the prepared primary antibodies, the cell culture plate was placed in a humid box and incubated overnight at 4 °C. The plate was set on a rocking shaker for three washes, each lasting 5 min, to remove excess primary antibodies. Subsequent to this, the appropriate secondary antibody was added and incubated at room temperature in a light-protected environment for 50 min. For nuclear counterstaining using DAPI, the cells in the 24-well plate were washed three times for 5 min each on a rocking shaker, within a PBS solution of pH 7.4. After a gentle drying step, a DAPI staining solution was added to the designated area, followed by an incubation of 10 min at room temperature in the absence of light. The sealing of the samples involved a three-time wash for 5 min each in a PBS solution of pH 7.4, after which 200 μL of anti-fade mounting medium was added to each well for sealing. Subsequently, images were captured using a fluorescence microscope, 5 fields of view were randomly selected from each group, and the fluorescence intensity was analyzed using ImageJ software [49]. The relative fluorescence intensity is the fluorescence intensity of the target protein/DAPI fluorescence intensity.

### 4.11. ROS Release Analysis in the Spinal Cord Neuronal Necrosis Model

For this experiment, a 10 µM concentration of DCFH-DA was utilized. The culture medium in the 24-well cell culture plate was carefully aspirated, and a suitable volume of diluted DCFH-DA was added. Each well received 500 μL of the solution. The plate was then incubated at 37 °C under low light conditions for 20 min, with occasional inversion and gentle mixing every 3 to 5 min to ensure optimal probe–cell interaction. Upon completion of the incubation period, the culture medium was aspirated, and the cells were washed three times with PBS for 5 min each time. Following the washes, fluorescence microscopy was employed to observe the cells, and images were captured for subsequent analysis. After performing ROS staining according to the above procedure, collect cells in centrifuge tubes, wash them with PBS three times, and perform flow cytometry detection before analyzing the results.

### 4.12. JC-1 Variation in the Spinal Cord Neuronal Necrosis Model

Following the aforementioned cell culturing, for each experimental group, a range of 1 × 10^5^ to 3 × 10^5^ cells were resuspended in 500 µL of cell culture medium. Subsequently, 0.5 µL of JC-1 staining working solution was added, and the mixture was gently inverted several times to ensure thorough mixing. The cell suspension was then placed in a cell culture incubator for 20 min. During this incubation period, a JC-1 staining buffer (1×) was prepared by diluting 5× JC-1 staining solution with 4 mL of distilled water per 1 mL of JC-1 solution. This buffer was kept on ice for subsequent use. After the completion of the cell suspension incubation, the mixture was centrifuged at 1000 rpm for 4 min under 4 °C conditions. The supernatant was discarded, and the cell pellet was resuspended in JC-1 staining buffer (1×) and subjected to another round of centrifugation under the same conditions. This process was repeated to ensure proper cell washing and resuspension in JC-1 staining buffer (1×). The resuspended cells were then subjected to analysis using appropriate instrumentation.

### 4.13. Protein Immunoblot Analysis of NLRP3 Pathway-Related Proteins

As described in Section 4.6, following the extraction of cellular proteins, protein immunoblot experiments were conducted to assess the expression profile of the relevant proteins.

### 4.14. Data Analysis

All data were subjected to statistical analysis using GraphPad Prism 8.0 software. The results are presented as mean ± standard error of the mean (SEM). Statistical significance was determined using one-way ANOVA. A *p*-value of less than 0.05 was considered statistically significant, * denoted *p* < 0.05, ** denoted *p* < 0.01, *** denoted *p* < 0.001, and **** denoted *p* < 0.0001. Different symbols were used to indicate statistical differences.

## 5. Conclusions

In conclusion, AKBA exerts its neuroprotective effects through the Nrf2/ROS/Casp-1 axis, leading to a reduction in neuronal pyroptosis. This reduction in pyroptosis results in decreased secretion of inflammatory cytokines IL-18 and IL-1β, ultimately mitigating spinal cord neuronal damage and safeguarding neuron viability. These findings suggest that AKBA holds promise in promoting spinal cord injury repair by protecting neuronal cells.

## Figures and Tables

**Table 1:** Antibody dilution table.

Antibodies	Concentration	Species	Catalog No.	Supplier
IL-1β	1:500	Rabbit	WLH3903	Wanleibio (Shenyang, China)
β-Actin	1:1000	Rabbit	bs-0061R	Bioss (Beijing, China)
NLRP3	1:1000	Rabbit	WL02635	Wanleibio (Shenyang, China)
Pro-IL-1β	1:500	Rabbit	WL02257	Wanleibio (Shenyang, China)
Caspase-1	1:500	Rabbit	WLH4550	Wanleibio (Shenyang, China)
GSDMD	1:1000	Rabbit	DF12275	Affinity (Liyang, China)
ASC	1:500	Rabbit	WL02462	Wanleibio (Shenyang, China)
IL-18	1:500	Rabbit	WL01127	Wanleibio (Shenyang, China)

**Table 2:** Primer list.

Genes	Primer Sequence (5′-3′)
*IL-1β*	(F) 5′-TTGAGTCTGCACAGTTCCCC-3′ (R) 3′-GTCCTGGGGAAGGCATTAGG-5′
*NLRP3*	(F) 5′-TGCATGCCGTATCTGGTTGT-3′ (R) 3′-ACCTCTTGCGAGGGTCTTTG-5′
*IL-18*	(F) 5′-AGGGCACAGCCTCTCAGTT-3′ (R) 3′-ACTCATCGTTGTGGGGACAG-5′
*Casp-1*	(F) 5′-CTGACAAGATCCTGAGGGCA-3′ (R) 3′-AACTTGAGGGAACCACTCGG-5′
*β-actin*	(F) 5′-AGGCATCCTGACCCTGAAGTAC-3′ (R) 3′-GAGGCATACAGGGACAACACAG-5′

## Data Availability

The data used to support the findings of this study are included within the article.
